# HuR Knockdown in MLO-Y4 Osteocyte-like Cells Elevates OPG Expression and Suppresses Osteoclastogenesis In Vitro

**DOI:** 10.3390/ijms27010430

**Published:** 2025-12-31

**Authors:** Ziqiu Fan, Hideki Kitaura, Aseel Marahleh, Abdulrahman Mousa, Fumitoshi Ohori, Alexandru Craevschi, Sherif Rashad, Hiroyasu Kanetaka

**Affiliations:** 1 Orthodontics and Dentofacial Orthopedics Department, Graduate School of Dentistry, Tohoku University, Sendai 980-8575, Miyagi, Japan; fan.ziqiu.q1@dc.tohoku.ac.jp (Z.F.); fumitoshi.ohori.b4@tohoku.ac.jp (F.O.); hiroyasu.kanetaka.e6@tohoku.ac.jp (H.K.); 2 Frontier Research Institute for Interdisciplinary Sciences, Tohoku University, Sendai 980-8578, Miyagi, Japan; craevschi.alexandru.p7@dc.tohoku.ac.jp; 3 Department of Neurosurgical Engineering, Graduate School of Biomedical Engineering, Tohoku University, Sendai 980-8575, Miyagi, Japan; abdulrahman_zahran@live.com (A.M.); sherif.mohamed.rashad.e3@tohoku.ac.jp (S.R.); 4 Department of Translational Neuroscience, Graduate School of Medicine, Tohoku University, Sendai 980-8575, Miyagi, Japan; 5 Division of Advanced Dental Science and Technology, Graduate School of Biomedical Engineering, Tohoku University, Sendai 980-8579, Miyagi, Japan; 6 Division of Interdisciplinary Co-Creation (ICC-Division), Liaison Center for Innovative Dentistry, Graduate School of Dentistry, Tohoku University, Sendai 980-8575, Miyagi, Japan

**Keywords:** HuR, osteocyte, OPG, RANKL, osteoclastogenesis, RNA stability

## Abstract

Bone remodeling is maintained through the coordinated actions of osteoblasts, osteoclasts, and osteocytes, among which osteocytes serve as major regulators of osteoclast-mediated bone resorption through the receptor activator of the nuclear factor-κB ligand (RANKL)–osteoprotegerin (OPG) signaling axis. While molecular signals regulating osteocytic RANKL-OPG expression are fairly understood, how post-transcriptional mechanisms impact osteocyte function remains poorly defined. HuR (human antigen R) encoded by *Elavl1* (embryonic lethal abnormal vision-like 1), a ubiquitously expressed RNA-binding protein, is known for stabilizing AU-rich element-containing transcripts involved in inflammatory and stress responses; however, its role in osteocyte-derived bone resorption is unknown. In this study, we examined the effect of HuR loss on osteocyte–osteoclastogenesis. Short hairpin RNA (shRNA)-mediated HuR knockdown in MLO-Y4 osteocyte-like cells resulted in a significant increase in *OPG* mRNA and its protein expression, whereas RANKL levels remained unchanged, leading to a significantly reduced RANKL/OPG ratio. Both co-culture and conditioned-medium assays demonstrated that HuR-deficient osteocytes produced a markedly diminished osteoclastogenic environment. Actinomycin D chase experiments showed no alteration in *OPG* mRNA decay kinetics, and RNA immunoprecipitation (RIP)-PCR failed to detect HuR–*OPG* interactions, indicating that HuR regulates OPG expression through indirect mechanisms rather than mRNA binding. These findings identify HuR as an indirect regulator of osteocyte-derived OPG expression that impacts osteoclast differentiation and reveal a previously unrecognized mechanism by which HuR contributes to bone remodeling.

## 1. Introduction

Bone remodeling is a dynamic process governed by the coordinated actions of osteoblasts, osteoclasts, and osteocytes. As one of the most abundant and long-lived bone cells, osteocytes function as central regulators of bone turnover by integrating mechanical, metabolic, and hormonal cues and communicating them to osteoblasts and osteoclasts through a repertoire of secreted factors [1,2,3,4]. Among these factors, the receptor activator of the nuclear factor κB ligand (RANKL)–osteoprotegerin (OPG) regulatory axis is an important determinant of osteoclast differentiation: RANKL promotes osteoclastogenesis by binding to its receptor RANK on osteoclast precursors, whereas OPG prevents this process by acting as a soluble decoy receptor [5,6,7,8,9]. Perturbations in the RANKL/OPG ratio shift the balance of bone remodeling and contribute to the disproportionate bone resorption that underlies skeletal disorders such as osteoporosis and inflammatory bone loss [10,11,12,13].

Osteoclasts come from monocyte-macrophage lineage precursors of hematopoietic origin. Their early growth and survival depend on macrophage colony-stimulating factor (M-CSF), which signals through its receptor c-Fms [14,15]. After this stage, osteoclast precursors differentiate into mature, bone-resorbing osteoclasts in response to RANKL, which binds its receptor RANK. This process is limited by OPG, a soluble decoy receptor that blocks RANKL [16,17,18,19,20]. The balance between M-CSF, RANKL, and OPG ultimately drives osteoclastogenesis.

RNA-binding proteins (RBPs) are key post-transcriptional regulators that modulate mRNA stability, localization, and translation, thereby shaping the translational output and proteomic landscape that govern bone remodeling [21,22,23]. Among RBPs, HuR (human antigen R) encoded by *Elavl1* (embryonic lethal abnormal vision-like 1) is a ubiquitously expressed RBP known for stabilizing AU-rich element–containing transcripts involved in immune signaling and stress responses [24,25]. HuR has been shown to directly bind and stabilize the AU-rich element of *TNF-α* mRNA, thereby enhancing cytokine expression at the post-transcriptional level [26]. A recent study using global and limb mesenchyme-specific deletion of *Elavl1* demonstrated that loss of HuR disrupts skeletal development, alters bone microarchitecture, and impairs osteoblast differentiation and matrix mineralization [27]. Consistent with these findings, integrated genomic and transcriptomic analyses have identified HuR as a regulatory gene within bone-related gene networks, and functional studies showed that HuR promotes osteogenic differentiation by stabilizing transcripts such as β-catenin and modulating the transcriptomic programs of bone-forming cells [28]. HuR is a key post-transcriptional regulator of stress- and inflammation-responsive gene programs [29,30]. We recently demonstrated that HuR regulates osteocyte stress responses by stabilizing *Txnip* mRNA, thereby modulating oxidative stress signaling [31]. Osteocyte-derived control of osteoclast differentiation is highly sensitive to cellular stress and inflammatory cues, particularly through the RANKL–OPG axis [32]. Therefore, we hypothesized that HuR may influence osteocyte-mediated regulation of osteoclastogenesis by modulating RANKL–OPG balance.

In our research, we identify HuR as a regulator of OPG expression in osteocytes. Using short hairpin RNA (shRNA)-mediated HuR knockdown in MLO-Y4 osteocyte-like cells, we found that HuR knockdown increased OPG expression and secretion without shifts in RANKL expression, resulting in a markedly reduced RANKL/OPG ratio. Functionally, HuR knockdown in osteocytes showed diminished ability to support osteoclastogenesis in co-culture and conditioned-medium (CM) systems. Importantly, *OPG* mRNA stability was not altered, suggesting that HuR influences *OPG* mRNA expression through an indirect mechanism. Together, these findings reveal a novel function of HuR in controlling osteocyte osteoclastogenesis which can impact bone remodeling and skeletal homeostasis in health and disease.

## 2. Results

### 2.1. Validation of HuR Knockdown in MLO-Y4 Cells

**Figure 1:**
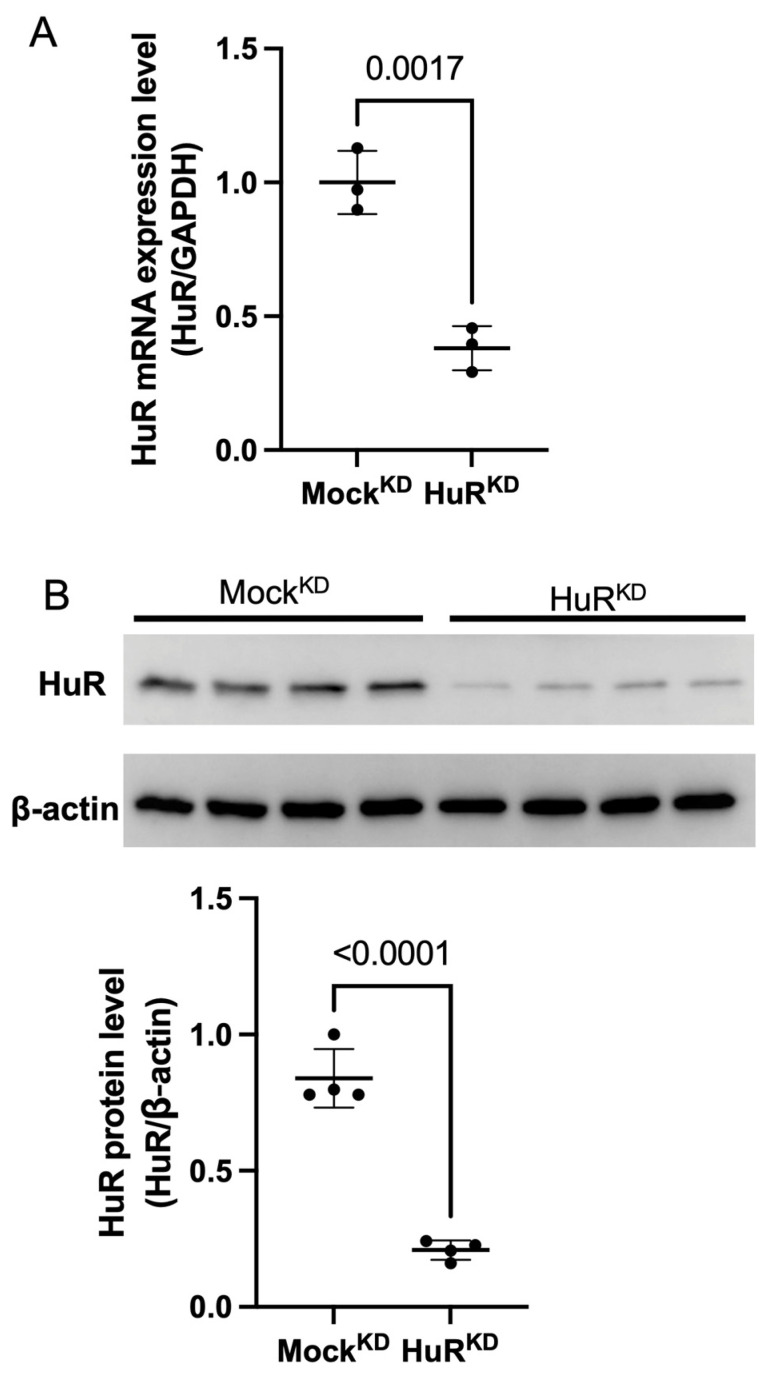
Validation of HuR knockdown (KD) in osteocyte-like MLO-Y4 cells. (**A**) mRNA expression of HuR after HuR^KD^ was analyzed by quantitative PCR. Data are presented as mean ± SD from three biologically independent experiments. (**B**) Western blot confirming reduced HuR protein expression in HuR^KD^ cells. Densitometric quantification of HuR band intensities normalized to β-actin is shown in the lower panel. Data are presented as mean ± SD from four biologically independent experiments.

To verify the shRNA-mediated HuR knockdown (KD) in osteocyte-like MLO-Y4 cells, we used a HuR-targeting shRNA (HuR^KD^), while a non-targeting shRNA served as the control (Mock^KD^). Quantitative PCR demonstrated that HuR transcript levels were reduced by approximately 62% compared with mock-transfected controls (Figure 1A). Consistently, Western blot analysis showed a corresponding decrease of about 75% in HuR protein abundance (Figure 1B). These findings confirm the effective knockdown of HuR expression.

### 2.2. HuR Knockdown Increases OPG Expression

**Figure 2:**
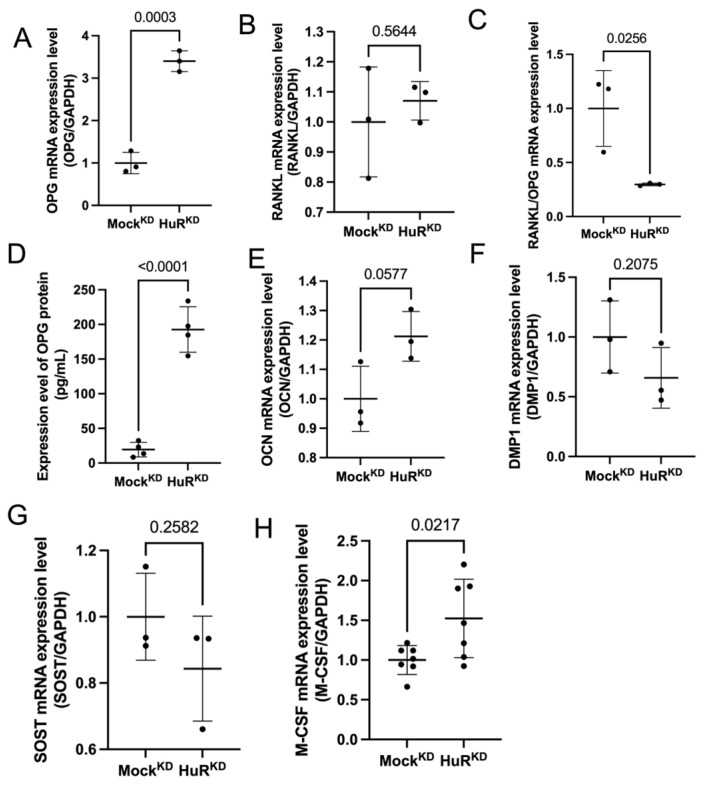
HuR knockdown increases OPG expression. (**A**) qPCR analysis showing that *OPG* mRNA expression was significantly elevated in HuR^KD^ cells compared with Mock^KD^. Data are presented as mean ± SD from three biologically independent experiments. (**B**) *RANKL* mRNA expression remained unchanged following HuR^KD^. Data are presented as mean ± SD from three biologically independent experiments. (**C**) The *OPG/RANKL* mRNA ratio was reduced in HuR^KD^ cells. Data are presented as mean ± SD from three biologically independent experiments. (**D**) ELISA measurement of OPG protein in culture supernatants revealed a significant increase in secreted OPG levels after HuR^KD^. Data are presented as mean ± SD from four biologically independent experiments. (**E**–**G**) Expression of other osteocyte-related markers, including *DMP1*, *OCN*, and *SOST*, showed no significant differences between HuR^KD^ and Mock^KD^ groups. Data are presented as mean ± SD from three biologically independent experiments. (**H**) Quantitative PCR analysis showing that *M-CSF* mRNA expression was significantly upregulated in the HuR^KD^ group compared with Mock^KD^. Data are presented as mean ± SD from seven biologically independent experiments.

To determine how HuR depletion affects osteocyte-derived signals, we analyzed the expression of a panel of osteocyte-associated transcripts in MLO-Y4 cells following shRNA-mediated HuR knockdown. Quantitative PCR revealed a robust increase in *Tnfrsf11b* (OPG) mRNA, whereas *Tnfsf11* (RANKL) expression remained unchanged (Figure 2A,B). Consistent with these transcriptional changes, the RANKL/OPG mRNA ratio was markedly reduced in HuR^KD^ cells (Figure 2C). ELISA measurements further confirmed a significant increase in secreted OPG protein in conditioned medium collected from HuR^KD^ MLO-Y4 cells (Figure 2D).

To determine whether HuR loss globally alters osteocytic identity or selectively affects specific cytokines, we examined a broader set of osteocyte-related markers. The expression of *Sost* (SOST), *Dmp1* (DMP1), and *Bglap* (osteocalcin; OCN) showed no significant differences between groups (Figure 2E–G). In contrast, *Csf1* (M-CSF) expression exhibited a modest but significant increase in HuR^KD^ cells (Figure 2H), although this upregulation did not translate into enhanced osteoclastogenesis in functional assays (see Section 2.3).

Collectively, these data demonstrate that HuR knockdown exerts a selective regulatory effect, most notably by increasing OPG expression and reducing the RANKL/OPG ratio, an essential determinant of osteoclast differentiation.

### 2.3. HuR Knockdown Reduces Osteoclast Formation in Co-Culture and Conditioned-Medium Culture

**Figure 3:**
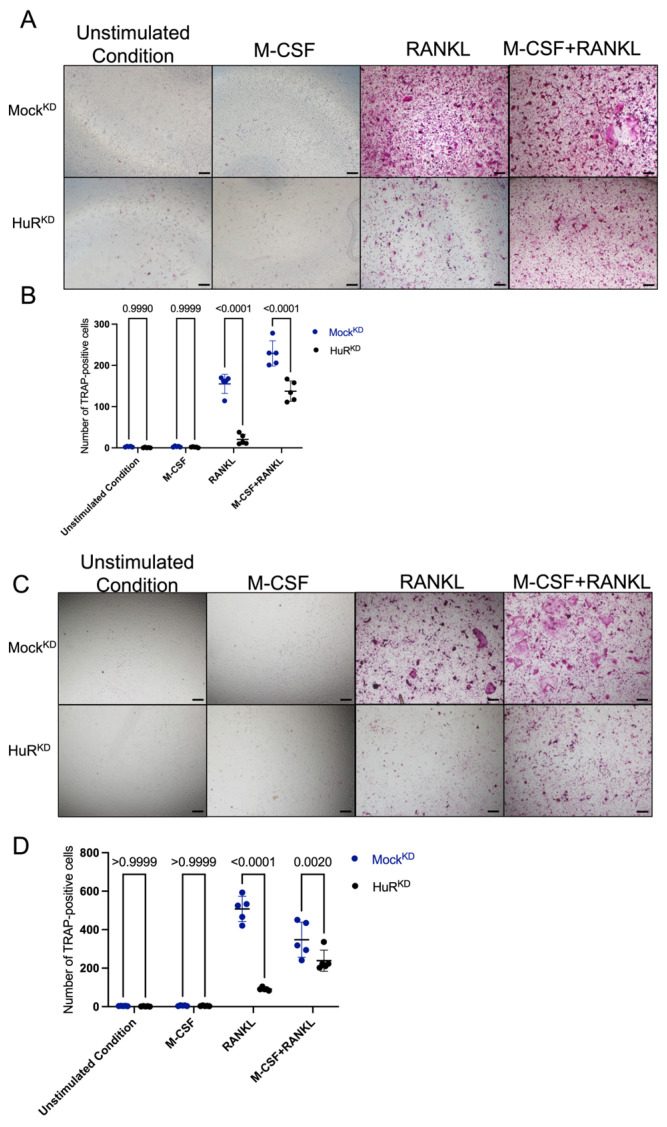
HuR knockdown in osteocytes reduces osteoclast formation in co-culture and conditioned-medium (CM) systems. (**A**) Representative TRAP staining images of osteoclasts formed in co-culture with Mock^KD^ or HuR^KD^ under different stimulation conditions (Unstimulated condition, M-CSF, RANKL, and M-CSF + RANKL). Scale bar = 200 μm. (**B**) Quantification of TRAP-positive multinucleated cells per well. (**C**) Representative TRAP staining images of osteoclasts formed in CM derived from Mock^KD^ or HuR^KD^ cells under different stimulation conditions (Unstimulated condition, M-CSF, RANKL, and M-CSF + RANKL). Scale bar = 200 μm. (**D**) Quantification of TRAP-positive multinucleated cells per well. Data are presented as mean ± SD from five biologically independent experiments.

To determine the functional consequence of the reduced RANKL/OPG ratio, we evaluated osteoclast formation using both co-culture and CM systems. In co-cultures, TRAP-positive multinucleated cells were not observed under unstimulated or M-CSF–only conditions, with no differences between groups. In contrast, RANKL stimulation induced osteoclastogenesis in Mock^KD^ co-cultures, whereas HuR^KD^ co-cultures displayed significantly fewer TRAP-positive multinucleated cells compared to Mock^KD^. A similar inhibitory effect of HuR knockdown was observed under combined M-CSF + RANKL stimulation (Figure 3A).

To isolate the contribution of osteocyte-derived secreted factors, we next examined CM-induced osteoclast differentiation. CM collected from HuR^KD^ osteocytes generated markedly fewer TRAP-positive osteoclasts than medium from Mock^KD^ cells under either RANKL stimulation or combined M-CSF and RANKL stimulation (Figure 3B).

Together, these findings demonstrate that HuR knockdown in osteocytes diminishes their ability to support osteoclastogenesis in both co-culture and CM models. The consistent reduction across co-culture and CM systems indicates that increased OPG production in HuR-deficient osteocytes plays a dominant inhibitory role in limiting RANKL-driven osteoclast formation.

### 2.4. OPG mRNA Stability Is Not Affected by HuR Depletion

**Figure 4:**
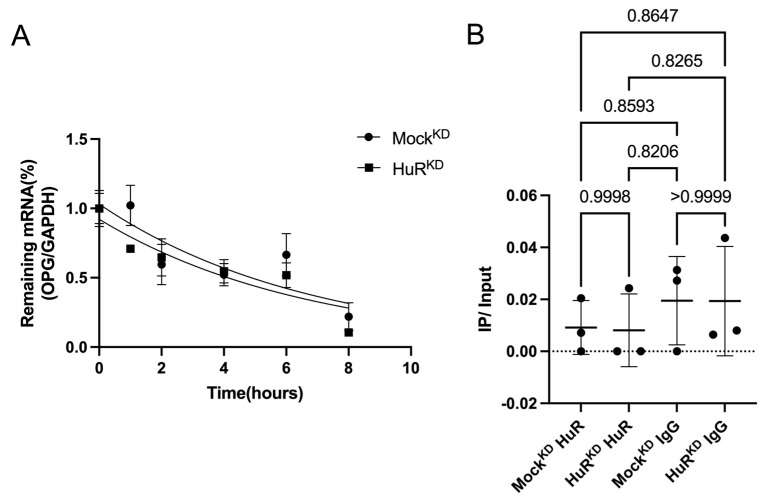
HuR knockdown does not alter *OPG* mRNA stability and *OPG* is not enriched in HuR immunoprecipitates. (**A**) Actinomycin D chase assay in MLO-Y4 cells with or without HuR knockdown. Cells were treated with actinomycin D (10 μg/mL), and *OPG* mRNA levels were quantified at the indicated time points (0–8 h) relative to GAPDH. Nonlinear regression using a one-phase decay model showed comparable decay kinetics between Mock^KD^ and HuR^KD^ cells (half-life ≈ 4.6 h; k = 0.148 h^−1^), indicating that HuR depletion does not affect *OPG* mRNA stability. Data are presented as mean ± SD from four biologically independent experiments. (**B**) RIP-PCR analysis assessing HuR binding to *OPG* mRNA. Lysates from Mock^KD^ and HuR^KD^ cells were subjected to immunoprecipitation using anti-HuR antibody or IgG control. *OPG* transcript levels in immunoprecipitates were normalized to input RNA. *OPG* was not enriched in HuR IP compared with IgG IP in either group, demonstrating that OPG is not a detectable HuR-binding target in osteocytes. Data are presented as mean ± SD from three biologically independent experiments.

To determine whether HuR regulates OPG expression through transcript stabilization, we performed an actinomycin D chase assay to evaluate *Tnfrsf11b* (OPG) mRNA decay. Nonlinear regression using a one-phase decay model showed nearly identical decay kinetics between Mock^KD^ and HuR^KD^ cells (half-life ≈ 4.6 h; k = 0.148 h^−1^), indicating that HuR knockdown does not alter *OPG* mRNA stability (Figure 4).

### 2.5. HuR Does Not Bind OPG mRNA in Osteocytes

To further examine whether HuR directly interacts with OPG transcripts, we performed RNA immunoprecipitation (RIP)-PCR using an anti-HuR antibody. *Tnfrsf11b* (OPG) mRNA was not enriched in HuR immunoprecipitates relative to IgG controls, and the signal remained at background levels in both Mock^KD^ and HuR^KD^ cells. These results demonstrate that *OPG* is not a detectable HuR-binding target in osteocytes (Figure 4B). Previous studies from our group have established that HuR can directly bind ARE-containing transcripts in osteocytes, such as *Txnip* mRNA, indicating that the RIP conditions used here are sufficient to detect bona fide HuR–mRNA interactions [31].

## 3. Discussion

**Figure 5:**
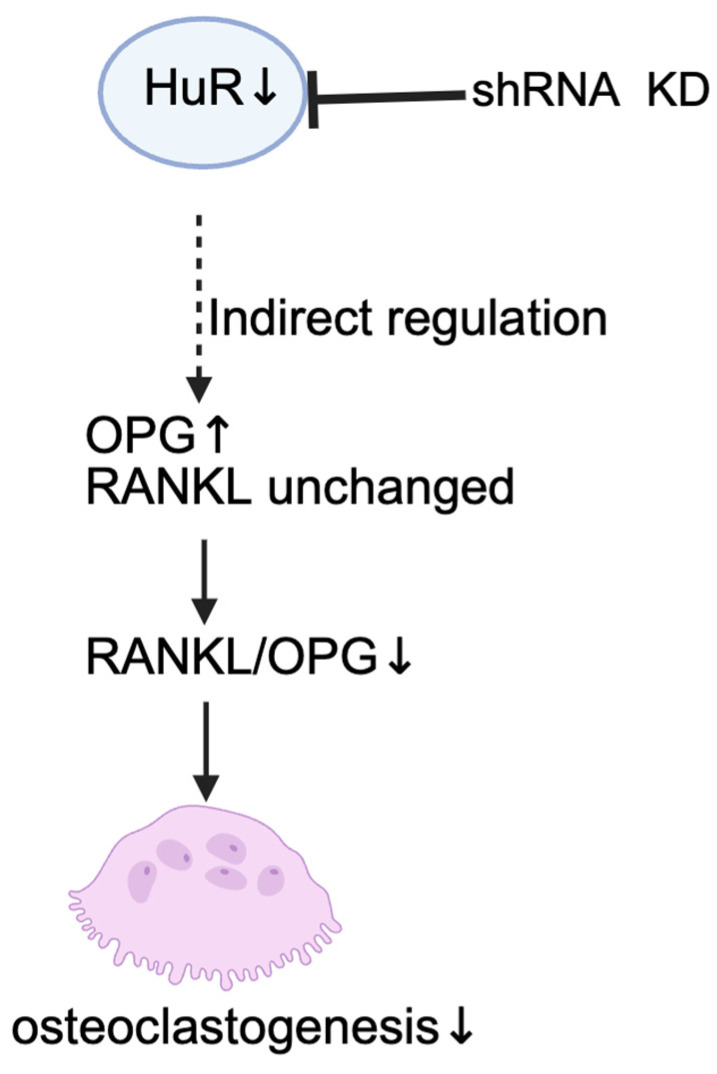
Schematic representation of how HuR knockdown in MLO-Y4 osteocyte-like cells alters osteoclastogenesis. Loss of HuR elevates OPG expression while leaving RANKL unchanged, resulting in a reduced RANKL/OPG ratio and decreased osteoclastogenesis. Symbols (↑/↓) indicate increase/decrease, respectively.

In this study, we show that HuR depletion in osteocyte-like MLO-Y4 cells under in vitro conditions leads to an increase in OPG expression at both the mRNA and protein levels, accompanied by a reduction in osteoclast formation in co-culture and conditioned-medium systems. Because *RANKL* mRNA remained unchanged, the reduced RANKL/OPG ratio explains diminished osteoclastogenesis (Figure 5).

HuR, a ubiquitously expressed RNA-binding protein, has been implicated in skeletal development. A study using Prx1-Cre–mediated deletion of HuR demonstrated that HuR is required for normal skeletal development and trabecular bone architecture, and that its loss impairs osteoblast differentiation and matrix mineralization [27]. Recent work has shown that HuR promotes osteoblast differentiation by stabilizing *LRP6* mRNA and enhancing Wnt/β-catenin signaling, and its overexpression alleviates osteoporotic phenotypes in ovariectomized mice [23]. HuR is well known for stabilizing AU-rich element–containing transcripts involved in cytokine signaling and stress adaptation. HuR has been shown to target mRNAs including TNF-α, COX2 and other inflammatory mediators [33,34,35], many of which influence bone turnover under physiological and disease conditions. HuR expression and activity are known to be modulated in pathological conditions characterized by inflammation and cellular stress. In inflammatory bone diseases such as rheumatoid arthritis, pro-inflammatory cytokines and stress signaling pathways have been reported to regulate HuR expression and subcellular localization, impacting skeletal homeostasis [36,37].

Based on increased mRNA levels of *OPG* upon HuR knockdown, we hypothesized that HuR might regulate OPG by stabilizing its mRNA. However, the actinomycin D chase assay revealed no difference in *OPG* mRNA decay kinetics between control and HuR-knockdown cells, supporting the conclusion that HuR does not directly regulate *OPG* mRNA stability under these experimental conditions. Additionally, RIP-PCR did not detect an interaction between HuR and *OPG* transcripts. These results indicate that OPG upregulation in HuR knockdown osteocytes is unlikely to result from stabilization of its mRNA, suggesting the involvement of indirect or upstream mechanisms regulating OPG expression. Previous studies have shown that HuR can influence gene expression indirectly without directly binding the target transcript; for example, HuR does not directly bind *IL-6* mRNA but alters IL-6 production by modulating the levels of another RNA-binding protein, tristetraprolin (TTP), which promotes *IL-6* mRNA decay [38]. This indicates that HuR can influence cytokine expression through other regulatory proteins instead of binding the target mRNA itself. A comparable indirect mechanism may also explain why OPG increases when HuR is depleted in osteocytes. These findings point to an indirect mode of regulation by HuR. One plausible hypothesis is that HuR activity regulates inflammatory cytokine expression, such as expression of IL-6 and TNF-α, which regulate OPG expression in osteocytes.

In addition to its effect on OPG, HuR depletion modestly increased *Csf1* (M-CSF) mRNA expression, whereas other osteocyte factors remained unchanged. Although M-CSF is required for the survival and proliferation of early osteoclast precursors, this increase did not translate into enhanced osteoclast formation in our conditioned-medium assays. This finding indicates that the increase in M-CSF expression alone was insufficient to promote further osteoclast formation under our experimental conditions.

In this study, osteoclastogenesis was evaluated primarily by TRAP staining and quantification of TRAP-positive multinucleated cells, which reflects osteoclast differentiation and formation. A limitation of this study is that osteoclast activity was inferred from formation-related readouts rather than directly assessed using resorption assays or late-stage functional markers such as Cathepsin K or NFATc1. As a result, our conclusions specifically address osteoclast formation, and future work will be required to determine how HuR-dependent osteocyte signaling influences osteoclast resorptive function.

Taken together, our findings indicate that HuR indirectly regulates osteocyte OPG expression in an in vitro osteocyte-like cell system, and that its depletion increases OPG transcription and translation and reduces osteoclast formation. These changes collectively shift the cytokine milieu toward an anti-resorptive state and attenuate osteoclast differentiation.

In conclusion, we identify HuR as an important modulator of osteocyte–osteoclast communication. Its role in indirectly regulating OPG expression highlights how RBPs regulate the balance of bone remodeling. These findings advance our understanding of skeletal homeostasis and suggest a potential avenue for targeting RBPs to treat unbalanced bone resorption.

## 4. Materials and Methods

### 4.1. Short Hairpin RNA Knockdown

To generate the HuR knockdown construct, two independent shRNA sequences targeting mouse *Elavl1* (HuR) were designed using the VectorBuilder shRNA design tool. The targeting sequences were as follows: shRNA construct A, 5′-CATTGGGAGAACGAATTTAAT-3′, and shRNA construct B, 5′-TTGTTAGTGTACAACTCATTT-3′. The synthesized oligonucleotides (FASMAC, Atsugi, Japan) were annealed and inserted into the pLKO.1-puro vector. An empty pLKO.1-puro plasmid (Addgene #8453) served as the non-targeting control. Correct insertion was verified by colony PCR and Sanger sequencing (Eurofins Genomics, Tokyo, Japan). Lentiviral particles were produced by co-transfecting HEK293T cells with the pLKO.1-shHuR plasmid, psPAX2 packaging plasmid (Addgene #12260), and pMD2.G envelope plasmid (Addgene #12259) using Lipofectamine 3000 (Thermo Fisher Scientific, Waltham, MA, USA). Viral supernatants were collected at 48 and 72 h, filtered (0.45 μm PVDF), and used immediately or stored at −80 °C. MLO-Y4 cells were transduced with the lentiviral supernatant (4:1 ratio with standard medium) containing 8 µg/mL polybrene (Sigma-Aldrich, St. Louis, MO, USA) for 24 h. After 6 h of recovery in standard medium, puromycin (2 µg/mL) was added for selection. Cells were maintained under selection for 7 days before use in subsequent experiments. Both shRNA constructs were initially evaluated for HuR knockdown efficiency and specificity. One construct demonstrated robust and reproducible suppression of HuR expression, whereas the other showed evidence of off-target effects and was therefore excluded from further analyses [31]. HuR knockdown efficiency of the selected construct was confirmed by RT-qPCR and Western blot analysis. The construct showing robust suppression of HuR expression was used in all subsequent experiments. Lentiviral transduction was performed once to establish a stable HuR knockdown cell population, which was subsequently used for all downstream experiments.

### 4.2. Real-Time Quantitative Polymerase Chain Reaction (qPCR)

Total RNA was extracted from cultured cells using the RNeasy Mini Kit (QIAGEN, Hilden, Germany) in accordance with the manufacturer’s instructions. Complementary DNA (cDNA) was synthesized from total RNA using the SuperScript IV First-Strand Synthesis System (Invitrogen, Carlsbad, CA, USA) according to the manufacturer’s instructions. RNA concentration and purity were assessed using a NanoPhotometer N60 (Implen, Munich, Germany), and samples with A260/280 ratios between 1.8 and 2.0 were used for subsequent analyses. Quantitative PCR was conducted on a CFX96 Touch Real-Time PCR Detection System (Bio-Rad Laboratories, Hercules, CA, USA). Gene expression levels were analyzed using the ΔCt method after normalization to GAPDH. The following primers were used in this study: GGTGGAGCCAAAAGGGTCA-3′ and 5′-GGGGGCTAAGCAGTTGGT-3′ for GAPDH; 5′-ATCAGAGCCTCATCACCTT-3′ and 5′-CTTAGGTCCAACTACAGAGGAAC-3′ for OPG; 5′-CCTGAGGCCCAGCCATTT-3′ and 5′-CTTGGCCCAGCCTCGAT-3′ for RANKL; 5′-TGATTGGGAATGGACACCTG-3′ and 5′-AAAGGCAATCTGGCATGAAGT-3′ for M-CSF; 5′-ACCACACGGACAGCAGTGAATC-3′ and 5′-CCTCATCGCCAAAGGTATCATCTC-3′ for DMP1; 5′-AGCCTTCAGGAATGATGCCAC-3′ and 5′-CTTTGGCGTCATAGGGATGGT-3′ for SOST; and 5′-CGCTCTGTCTCTCTGACCTC-3′ and 5′-GACTGAGGCTCCAAGGTAGC-3′ for OCN.

### 4.3. Preparation of Osteoclast Precursors

Osteoclast precursors were generated from bone marrow cells isolated from 8–10-week-old male C57BL/6 mice (CLEA Japan, Inc., Tokyo, Japan). Briefly, femurs and tibiae were excised and cleaned of soft tissue, then, bone marrow cells were collected by centrifugation of the bones α-Minimum Essential Medium (α-MEM; Sigma-Aldrich) at 900× *g*. The resultant cell suspension was passed through a 40 μm nylon cell strainer (Falcon, Corning, NY, USA) and centrifuged at 1500× *g* twice at 4 °C. Cells were then seeded into 10 cm culture dishes and cultured in α-MEM supplemented with 10% fetal bovine serum (FBS), 100 IU/mL penicillin G, and 100 μg/mL streptomycin (Meiji Seika, Tokyo, Japan). To generate osteoclast precursors, bone marrow-derived cells were cultured in the presence of M-CSF, 100 ng/mL derived from the CMG14-12 cell line for 3 days, yielding M-CSF-dependent macrophages [39].

### 4.4. Co-Culture of Osteocyte-like Cells and Osteoclast Precursors

To assess the modulatory effect of osteocytic HuR on osteoclast differentiation, Mock^KD^ or HuR^KD^ cells were seeded in 96-well plates at a density of 500 cells/well. Osteoclast precursors obtained as described above were then added at 3 × 10^4^ cells/well in α-MEM supplemented with 10% FBS and 100 IU/mL penicillin–streptomycin. The co-culture was stimulated with M-CSF (10 ng/mL), RANKL (10 ng/mL), or a combination of both cytokines, while basal wells received no stimulation. Media were replaced every 2 days. After 5 days, cells were fixed with 10% neutral buffered formalin and permeabilized with 0.2% Triton X-100. Tartrate-resistant acid phosphatase (TRAP) staining was performed as previously described, and TRAP-positive multinucleated cells (≥3 nuclei) were counted under a light microscope.

### 4.5. Conditioned-Medium-Induced Osteoclast Differentiation

For conditioned-medium (CM) experiments, Mock^KD^ or HuR^KD^ cells were cultured until 80–90% confluence. The collected supernatants were centrifuged at 1500× *g* for 5 min and filtered through a 0.22 μm membrane to remove debris, generating osteocyte-conditioned media. Osteoclast precursors (3 × 10^4^ cells/well) were cultured in 96-well plates and cultured in a 1:1 mixture (by volume) of fresh α-MEM supplemented with 10% fetal bovine serum and osteocyte-conditioned medium. Fresh medium contained M-CSF at 50 ng/mL, resulting in a final concentration of 25 ng/mL M-CSF in the culture system. Where indicated, RANKL was added to the fresh medium at 50 ng/mL, yielding a final concentration of 25 ng/mL after mixing with conditioned medium. The CM system included the following conditions: unstimulated CM, CM supplemented with M-CSF alone, CM supplemented with RANKL alone, and CM supplemented with both M-CSF and RANKL. After 5 days, TRAP staining and quantification were performed as in the co-culture experiments.

### 4.6. Enzyme-Linked Immunosorbent Assay (ELISA)

Secreted OPG protein levels were measured using a Mouse Osteoprotegerin/TNFRSF11B ELISA Kit (KE10058, Proteintech, Rosemont, IL, USA) according to the manufacturer’s protocol. Culture supernatants were collected from Mock^KD^ and HuR^KD^ MLO-Y4 cells at comparable confluence (80–90%) under identical culture conditions, centrifuged at 1500× *g* for 5 min to remove cell debris, and stored at −20 °C until analysis. On the day of assay, samples were thawed on ice and, when necessary, diluted in the sample diluent provided with the kit. A series of OPG standards was prepared to generate a standard curve, and 100 µL of standards and samples were added to antibody-precoated 96-well plates in duplicate. After incubation and washing steps, bound OPG was detected with the HRP-conjugated detection antibody and TMB substrate. The reaction was stopped with acidic stop solution, and absorbance was read at 450 nm with a 570 nm reference using a microplate reader. OPG concentrations were calculated from a four-parameter logistic standard curve and expressed as pg/mL.

### 4.7. Western Blot

Cells were lysed on ice for 20 min in RIPA buffer (Millipore, Burlington, MA, USA) supplemented with a protease and phosphatase inhibitor cocktail (Thermo Fisher Scientific, Waltham, MA, USA). Lysates were clarified by centrifugation at 14,000× *g* to remove insoluble material. Total protein concentration was measured using the Pierce™ BCA Protein Assay Kit (Thermo Fisher Scientific). Protein samples were mixed with Laemmli sample buffer and β-mercaptoethanol (Bio-Rad Laboratories, Hercules, CA, USA) at a 3:1 ratio and denatured at 95 °C for 5 min. Equal amounts of protein were resolved on 4–15% Mini-PROTEAN TGX precast gels (Bio-Rad) and electrotransferred to PVDF membranes using a Trans-Blot Turbo Transfer System (Bio-Rad). β-actin was used as the loading control. Membranes were blocked in Block-Ace solution (DS Pharma Biomedical, Osaka, Japan) for 1 h at room temperature and incubated overnight at 4 °C with primary antibodies against HuR (1:1000, Abcam, Cambridge, UK; cat.no. ab200342) and β-actin (1:5000, Sigma-Aldrich, cat.no. A2228). After washing with TBS-T, membranes were probed for 1 h at room temperature with HRP-conjugated anti-rabbit (1:5000; Cell Signaling Technology, Danvers, MA, USA) or anti-mouse (1:10,000; GE Healthcare, Chicago, IL, USA) secondary antibodies. Protein bands were visualized using SuperSignal™ West Femto substrate (Thermo Fisher Scientific) and imaged with a FUSION-FX6 EDGE Chemiluminescence System (Vilber Lourmat, Collégien, France).

### 4.8. mRNA Stability Assay

To determine the half-life of specific mRNAs, transcriptional activity was inhibited by adding Actinomycin D (ActD, final concentration 10 µg/mL) to the culture medium. Cells were collected at 0, 1, 2, 4, 6, and 8 h after ActD treatment, rapidly rinsed with ice-cold PBS, and lysed in TRIzol reagent (Invitrogen, Carlsbad, CA, USA) for RNA extraction. Subsequent cDNA synthesis and quantitative PCR were performed as described above. The remaining mRNA levels at each time point were normalized to baseline values (t = 0 h) and plotted to generate decay curves. The degradation constant (k) and mRNA half-life (t_1_/_2_) were calculated by nonlinear regression using a one-phase exponential decay model in GraphPad Prism v9.5.1 (GraphPad Software, San Diego, CA, USA). The quality of curve fitting was assessed by the coefficient of determination (R^2^) using an ordinary least-squares fit at a 95% confidence interval.

### 4.9. RNA Immunoprecipitation (RIP) Assay

RNA immunoprecipitation (RIP) was carried out using the EZ-Magna RIP RNA-Binding Protein Immunoprecipitation Kit (Millipore, Billerica, MA, USA) following the protocol supplied by the manufacturer. In brief, MLO-Y4 cells grown to approximately 80% confluence were harvested and lysed in the kit’s RIP lysis buffer. An aliquot of 100 µL clarified lysate was combined with RIP buffer containing magnetic beads pre-bound to either an anti-HuR antibody, normal rabbit IgG (negative control), or anti-SNRNP70 (kit positive control). A fraction of the clarified lysate was reserved as the input control prior to immunoprecipitation. RIP-qPCR enrichment was quantified using the IP/Input method and presented as the ratio of target transcript levels in the immunoprecipitated RNA relative to the corresponding input RNA. After incubation and sequential washing steps, the RNA–protein complexes captured on the beads were released, treated with proteinase K to remove proteins, and the associated RNA was isolated using the purification components included in the kit. The recovered RNA was subsequently reverse-transcribed and analyzed by RT-qPCR as outlined in the RT-qPCR section.

### 4.10. Statistical Analysis

All data are presented as mean ± standard deviation (SD). Student’s *t*-test was used to compare differences between two groups, and for multiple comparisons, one-way analysis of variance (ANOVA) followed by the Tukey–Kramer test was performed. Statistical significance was set at *p* < 0.05. All experiments were independently performed at least three times using biologically independent samples to ensure reproducibility. Technical replicates were averaged for statistical analysis.

## 5. Conclusions

In conclusion, this study identifies HuR as an important post-transcriptional regulator of osteocyte-mediated osteoclastogenesis. HuR depletion in MLO-Y4 osteocyte-like cells resulted in a consistent increase in OPG expression without altering RANKL levels, leading to a markedly reduced RANKL/OPG ratio and a diminished capacity to support osteoclast formation in both co-culture and conditioned-medium systems. Mechanistically, neither actinomycin D chase assays nor RIP-PCR revealed evidence that HuR directly binds OPG mRNA, indicating that the observed OPG upregulation occurs through indirect pathways. Together, these findings support a role for HuR in regulating osteocyte-derived osteoclastogenesis through indirectly regulating OPG expression.

## Data Availability

The original contributions presented in this study are included in the article. Further inquiries can be directed to the corresponding authors.
